# Prediction Models for Late-Onset Preeclampsia: A Study Based on Logistic Regression, Support Vector Machine, and Extreme Gradient Boosting Models

**DOI:** 10.3390/biomedicines13020347

**Published:** 2025-02-03

**Authors:** Yangyang Zhang, Xunke Gu, Nan Yang, Yuting Xue, Lijuan Ma, Yongqing Wang, Hua Zhang, Keke Jia

**Affiliations:** 1 Department of Clinical Laboratory, Peking University Third Hospital, Beijing 100191, China; zyy18813017610@163.com (Y.Z.);; 2 Institute of Hematology and Blood Diseases Hospital, Chinese Academy of Medical Sciences and Peking Union Medical College, Tianjin 300020, China; 3 Department of Obstetrics and Gynecology, Peking University Third Hospital, Beijing 100191, China; guxunke@163.com; 4 Department of Blood Transfusion, Peking University Third Hospital, Beijing 100191, China; 5 Research Center of Clinical Epidemiology, Peking University Third Hospital, Beijing 100191, China

**Keywords:** late-onset preeclampsia, maternal risk factors, laboratory indicators, logistic regression, support vector machine, extreme gradient boosting

## Abstract

**Background:** Preeclampsia, affecting 2–4% of pregnancies worldwide, poses a substantial risk to maternal health. Late-onset preeclampsia, in particular, has a high incidence among preeclampsia cases. However, existing prediction models are limited in terms of the early detection capabilities and often rely on costly and less accessible indicators, making them less applicable in resource-limited settings. **Objective:** To develop and evaluate prediction models for late-onset preeclampsia using general information, maternal risk factors, and laboratory indicators from early gestation (6–13 weeks). **Methods:** A dataset of 2000 pregnancies, including 110 late-onset preeclampsia cases, was analyzed. General information and maternal risk factors were collected from the hospital information system. Relevant laboratory indicators between 6 and 13 weeks of gestation were examined. Logistic regression was used as the baseline model to assess the predictive performance of the support vector machine and extreme gradient boosting models for late-onset preeclampsia. **Results**: The logistic regression model, only considering general information and risk factors, identified 19.1% of cases, with a false positive rate of 0.4%. When selecting 15 factors encompassing general information, risk factors, and laboratory indicators, the false positive rate increased to 0.7% and the detection rate improved to 27.3%. The support vector machine model, only considering general information and risk factors, achieved a detection rate of 27.3%, with a false positive rate of 0.0%. After including all the laboratory indicators, the false positive rate increased to 7.7% but the detection rate significantly improved to 54.5%. The extreme gradient boosting model, only considering general information and risk factors, achieved a detection rate of 31.6%, with a false positive rate of 1.5%. After including all the laboratory indicators, the false positive rate remained at 0.7% but the detection rate increased to 52.6%. Additionally, after adding the laboratory indicators, the areas under the ROC curve for the logistic regression, support vector machine, and extreme gradient boosting models were 0.877, 0.839, and 0.842, respectively. **Conclusion:** Compared with the logistic regression model, both the support vector machine and extreme gradient boosting models significantly improved the detection rates for late-onset preeclampsia. However, the support vector machine model had a comparatively higher false positive rate. Notably, the logistic regression and extreme gradient boosting models exhibited high negative predictive values of 99.3%, underscoring their effectiveness in accurately identifying pregnant women less likely to develop late-onset preeclampsia. Additionally, logistic regression showed the highest areas under the ROC curve, suggesting that the traditional model has unique advantages in relation to prediction.

## 1. Introduction

Preeclampsia (PE) is a specific hypertensive disorder that occurs during pregnancy, affecting approximately 2 to 4% of pregnancies worldwide [1]. It represents a leading cause of adverse pregnancy outcomes and perinatal mortality. Preeclampsia is commonly categorized into two types: early-onset preeclampsia, occurring before 34 weeks of gestation, and late-onset preeclampsia, occurring at or after 34 weeks of gestation. Studies investigating placental pathology in women with preeclampsia have identified significant distinctions between early-onset and late-onset PE. Early-onset PE is characterized by vascular lesions and placental insufficiency, whereas late-onset PE is associated with placental overgrowth [2]. Given the potential differences in the underlying mechanisms between early-onset and late-onset PE, researchers have advocated for separate investigations of these subtypes [3,4].

Several prediction models have been developed for preeclampsia, with higher detection rates for early-onset PE and lower detection rates for late-onset PE. This discrepancy may arise from the fact that late-onset PE occurs later in pregnancy and often presents with milder symptoms compared to early-onset PE [5,6]. However, the incidence of late-onset PE is significantly higher than that of early-onset PE, with rates of 2.72% and 0.38%, respectively [3]. These findings underscore the importance of research focused specifically on late-onset PE.

The Fetal Medicine Foundation (FMF) introduced a classic prediction model for preeclampsia in early pregnancy, known as the triple test [7]. This model incorporates various indicators, including maternal factors, mean arterial pressure (MAP), uterine artery pulsatility index (UtA-PI), and maternal serum or plasma placental growth factor (PlGF). However, measuring the UtA-PI requires ultrasound examination, and PlGF testing can be costly, limiting the widespread adoption of the FMF model in China. Therefore, there is a crucial need to explore prediction models for PE that are cost-effective and widely accessible. As a result, researchers have increasingly focused on the combination of maternal factors and clinical laboratory indicators.

In recent years, machine learning models have advanced rapidly, demonstrating their capacity to handle numerous predictive factors [8]. This provides a viable approach for developing prediction models utilizing a broader range of clinical laboratory indicators and risk factors. Overcoming the limitations of traditional logistic regression, which is constrained by the one-tenth principle of events per variable (EPV) [9], support vector machine (SVM) [10] and extreme gradient boosting (XGBoost) [11] have been employed in the investigation of preeclampsia prediction models. These models comprehensively consider maternal risk factors, clinical laboratory indicators, and more, exhibiting strong predictive performance across multiple experimental datasets.

In this study, we employed two machine learning algorithms, SVM and XGBoost, in addition to traditional logistic regression as the baseline model. These models were utilized to integrate and analyze maternal risk factors and clinical laboratory indicators. The objective was to establish an accurate and robust predictive model for late-onset PE, thereby providing more effective predictions and timely prevention strategies for maternal health.

## 2. Materials and Methods

### 2.1. Materials and Data Collection

**Figure 1:**
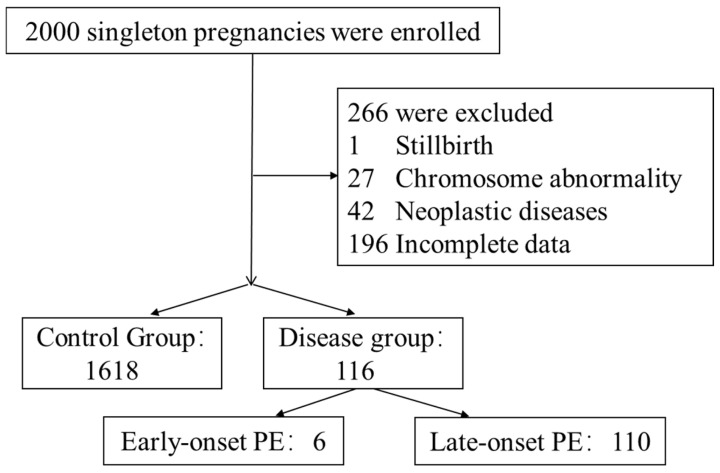
The number and classification of pregnant women included in this study.

This prospective study included a total of 2000 Chinese pregnant women, aged 18 to 46 years, who underwent routine prenatal examinations at the Department of Obstetrics and Gynecology, Peking University Third Hospital, from February 2021 to December 2021. Residual serum samples from routine biochemical tests during prenatal examinations at 6–13 weeks of gestation were collected. Blood collection required fasting, and only samples without hemolysis, jaundice, or lipemia were selected. These samples were then centrifuged at 2793× *g* for 10 min, and the resulting serum was stored at −80 °C. Additionally, general clinical information on the pregnant women was recorded, and their pregnancy progress was dynamically tracked. Finally, 110 cases of late-onset preeclampsia, which occurring at or after 34 weeks of gestation, and 1618 control cases were selected (Figure 1). The study protocol was approved by the Medical Science Research Ethics Committee of Peking University Third Hospital (IRB 00006761-M2021032).

#### 2.1.1. Inclusion and Exclusion Criteria

##### Inclusion Criteria

PE group: This group consisted of singleton pregnancies that developed late-onset preeclampsia. The diagnosis of preeclampsia was based on the diagnostic criteria outlined in ACOG practice bulletin No. 222 [12]. Late-onset preeclampsia was defined as the occurrence of preeclampsia at or after 34 weeks of gestation.

Control group: This group comprised singleton pregnancies without a history of significant illness and without any severe diseases during pregnancy.

##### Exclusion Criteria

Excluded from this study were cases involving fatal chromosomal abnormalities, stillbirth occurring before 24 weeks of gestation, malignant tumors, infectious diseases such as AIDS and hepatitis, and incomplete data.

### 2.2. Establishment of the Database

General information about the pregnant women, including their age, pre-pregnancy BMI, and mean arterial pressure (MAP), was obtained between 6 and 13 weeks of gestation through the hospital information system. Maternal risk factors that have been shown to increase the risk of preeclampsia were also collected, including chronic hypertension, diabetes, systemic lupus erythematosus, antiphospholipid syndrome, chronic kidney disease, assisted reproductive technology, and nulliparity [12].

Based on the pathophysiological characteristics of preeclampsia [13], specific indicators were identified for detection and collection during the initial phase of our research. These indicators encompassed renal function, liver function, lipid profile, iron metabolism, blood cell counts, complement levels, inflammation markers, as well as fibrinogen (Fn), homocysteine (HCY), and D-dimer. Routine laboratory indicators, such as liver function, renal function, lipid levels, and proteinuria, were obtained by reviewing the hospital information system records of pregnant women at 6–13 weeks of gestation. Proteinuria was defined as urinary protein 1+ or higher, or 24 h urinary protein levels ≥150 mg. Other indicators, such as complement C3, C4, factor B, factor H, and Fn, were measured using a biochemical analyzer (Beckman-Coulter Inc., Brea, CA, USA, AU5800).

The pregnant women were followed up during the pregnancy and information was obtained on their pregnancy outcomes and the occurrence of preeclampsia. The collected data, including general information on the pregnant women, maternal risk factors, laboratory indicators from 6–13 weeks of gestation, and the occurrence of late-onset PE, were organized to establish the database.

### 2.3. Statistical Analysis

The preliminary data analysis was conducted using IBM SPSS Statistics 25.0.0.0. The normality of the quantitative variables was assessed using the Kolmogorov–Smirnov test. Normally distributed variables were described as the mean ± standard deviation (mean ± SD), while non-normally distributed variables were described as the median (25th percentile, 75th percentile) (median [P25, P75]). Categorical variables were presented as the counts (percentages). For non-normally distributed variables, missing values were imputed using the median, while for normally distributed variables, the mean was used for imputation. Group comparisons were performed using *t* tests for normally distributed data, Mann–Whitney U tests for skewed data, and chi-square tests for binary categorical data. All the statistical tests were two-tailed, and a significance level of *p* < 0.05 was considered statistically significant. Data preprocessing involved replacing values outside the range of (mean − 3SD, mean + 3SD) with mean ± 3 × SD and standardizing quantitative variables using z-score normalization.

#### 2.3.1. Logistic Regression Model

Logistic regression is a widely used statistical modeling technique for classification problems. Its purpose is to predict the probability of a binary outcome (such as whether a patient will develop late-onset preeclampsia) based on various input variables. In our study, we utilized IBM SPSS Statistics 25.0.0.0 software to perform the logistic regression analysis.

Logistic regression analyzes the relationship between various predictors (such as demographic information and known risk factors) and the likelihood of developing a condition like late-onset preeclampsia. Unlike linear regression, which predicts a continuous outcome, logistic regression predicts the probability of an event occurring, with output values ranging between 0 and 1.

Initially, only general information and maternal risk factors were included in the analysis. Subsequently, laboratory indicators from 6 to 13 weeks of gestation were incorporated to enhance the model’s accuracy. A backward elimination method was employed to select relevant indicators, ensuring that only those with significant clinical relevance and minimal multicollinearity were retained. The final model included 15 variables, all of which had a significance level of *p* < 0.05.

#### 2.3.2. Support Vector Machine (SVM) Model

SVM is a powerful supervised learning method commonly used for classification and regression problems. Introduced by Vapnik et al. in 1992 [14], SVM is based on the principle of structural risk minimization. The core idea behind SVM is to find a decision boundary, called a hyperplane, that best separates different classes while maximizing the margin between them. The larger the margin, the better the model’s generalization ability.

SVM employs kernel functions to transform the original feature space into a higher-dimensional space, which allows it to handle complex relationships in the data. This transformation is essential for solving problems that cannot be separated by a simple straight line (linear problems). Commonly used kernel functions include linear, polynomial, radial basis function (RBF), and sigmoid kernels. Among these, the RBF kernel is particularly powerful for handling nonlinear problems, as it can capture intricate patterns that a linear model cannot.

In this study, we utilized IBM SPSS Modeler Subscription 18.0 software to build the SVM model. The study population was randomly divided into an 80% training set and a 20% test set. Initially, the model was trained using only maternal risk factors and general information. We explored different kernel functions and parameters to optimize the model’s performance. Based on the results, the RBF kernel with the parameters c = 8 and gamma = 0.01 was selected as the optimal choice for the model.

To evaluate the model’s performance, we used five-fold cross-validation, a technique that splits the data into five subsets, training the model on four subsets and testing it on the remaining one. This process was repeated five times to ensure the model’s robustness and generalization ability. The RBF kernel with the specified parameters was found to be the best option for our model, providing strong predictive performance.

#### 2.3.3. Extreme Gradient Boosting (XGBoost) Model

XGBoost is an advanced ensemble learning algorithm introduced by Chen and Guestrin in 2016 [15], primarily used for classification and regression tasks. It is based on the gradient boosting decision tree framework, where new decision trees are added iteratively to correct the prediction errors made by the previous trees. In simple terms, each new tree attempts to improve the model by focusing on the mistakes made by earlier trees.

What sets XGBoost apart from traditional gradient boosting decision trees is its unique features, including regularization, which helps prevent overfitting; parallel computing capabilities, which speed up the model training process; and an early stopping strategy, which halts the training process when further iterations no longer improve the model performance. These advantages make XGBoost highly efficient, accurate, and resistant to overfitting, making it one of the most powerful algorithms in machine learning today.

In our study, we used R version 4.1.2 to train the XGBoost models. To address the class imbalance (where the control group was much larger than the preeclampsia group), we applied undersampling to the control group, reducing its sample size to 15% of the preeclampsia group’s size. The study population was randomly split into an 80% training set and a 20% testing set. The training set was used to iteratively train the model, with each round improving the model’s accuracy by adjusting the parameters. The optimal parameters were selected as follows: max.depth = 7, eta = 0.1, subsample = 0.9, colsample_bytree = 0.5, nrounds = 50, and objective = binary: logistic.

Initially, the model was built using only general information and risk factors. Later, laboratory indicators collected during weeks 6 to 13 of pregnancy were added to the model to improve its predictive capability.

## 3. Results

### 3.1. Clinical Features

Among the initial 2000 pregnant women, 266 were excluded from this study due to not meeting the inclusion criteria. Out of the remaining participants, a total of 116 women were diagnosed with preeclampsia, including 110 (94.8%) cases of late-onset preeclampsia, which occurred at or after 34 weeks of gestation, and 6 (5.2%) cases of early-onset preeclampsia, which occurred before 34 weeks of gestation. The 110 cases of late-onset preeclampsia were included in the preeclampsia group, while the control group consisted of the remaining 1618 pregnant women without preeclampsia or other severe complications (Figure 1).

### 3.2. Comparison of General Information and Risk Factors of Control Group and PE Group

Maternal age, systemic lupus erythematosus, and antiphospholipid syndrome showed no statistically significant difference (*p* > 0.05), while the remaining indicators showed a statistically significant difference (*p* < 0.05). Since systemic lupus erythematosus did not occur in the PE group, it was not included in the model development (Table 1).

### 3.3. Comparison of Laboratory Indicators at 6–13 Weeks of Gestation Between the Control and PE Groups

The TBA, TP, Alb, GLO, LDH, and D-Bil in the liver function indicators; urea, Cr, Ca, and P in the renal function indicators; CHO and ApoA1 in the blood lipid indicators; and C1q, PLT/Lym, sTfR, HCY, and D-dimer showed no statistically significant difference between the two groups. Significant differences were observed in the remaining indicators (Table 2).

### 3.4. Logistic Regression

**Figure 2:**
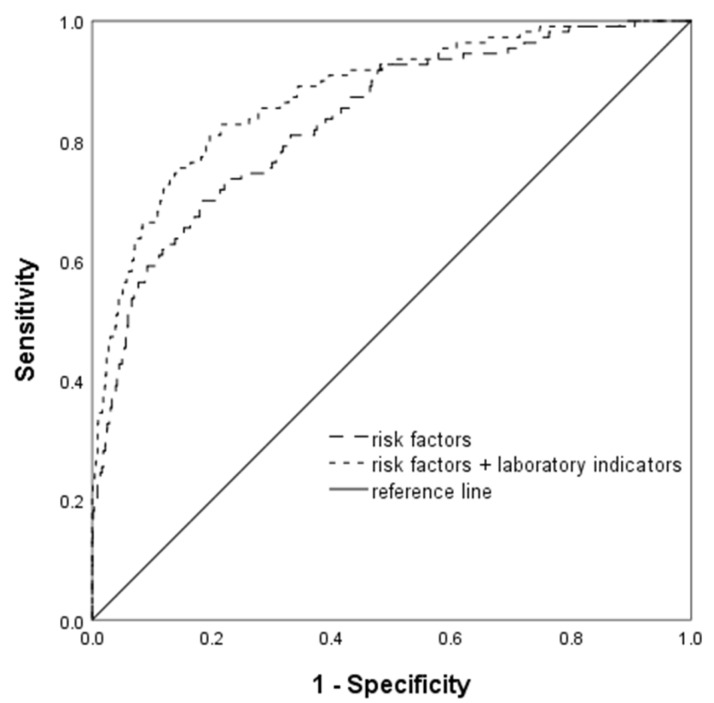
ROC curve of the logistic regression model.

Initially, a multivariable binary logistic regression analysis was conducted using only the general information and risk factors. At a 0.4% false positive rate (FPR), the detection rate was 19.1% and the area under the ROC curve (AUC^ROC^) was 0.833. Detailed information about the model can be found in Table 3. Next, we incorporated the laboratory indicators at 6–13 weeks of gestation and built the logistic regression model. The backward elimination method was applied to select indicators, taking into account their clinical significance and the presence of multicollinearity. Ultimately, 15 indicators with a significance level of *p* < 0.05 were retained. At a 0.7% FPR, the detection rate increased to 27.3% and the AUC^ROC^ was 0.877 (Table 4). The receiver operating characteristic (ROC) curve for the logistic regression model is presented in Figure 2.

### 3.5. SVM Model

**Figure 3:**
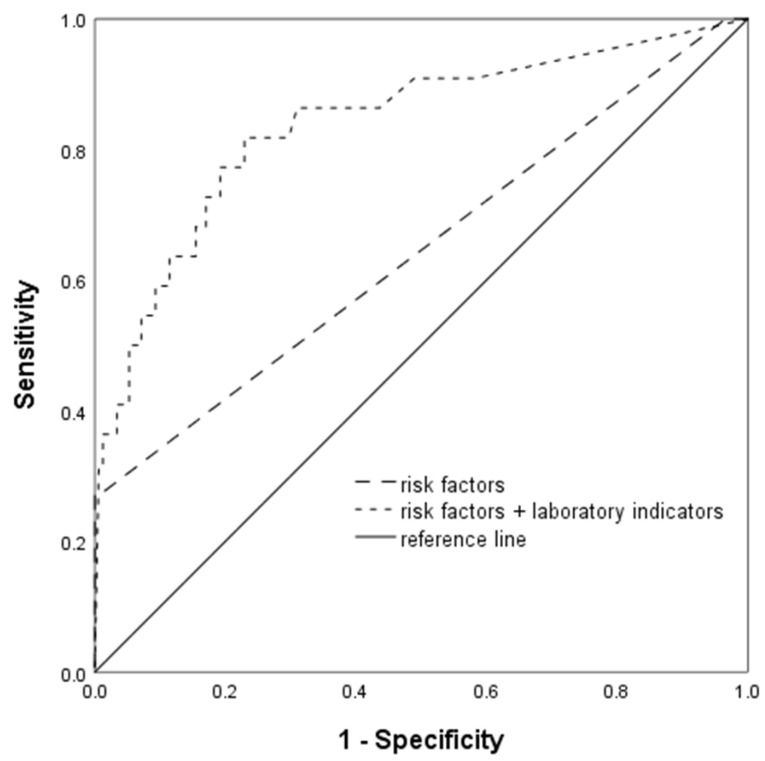
ROC curve of the SVM model.

The model was trained with the risk factors and general information and tested on the validation set. At an FPR of 0.0%, the detection rate was 27.3% and the AUC^ROC^ was 0.645. Subsequently, the laboratory indicators, along with the risk factors and general information, were used for modeling and testing. The five-fold cross-validation showed an overall accuracy of 90.8%. The top 18 indicators ranked by their importance in the model were as follows: MAP, chronic hypertension, UIBC, kidney disease, proteinuria, BMI, TIBC, HDL-C, ApoA1, ALB, Fn, C4, CHO, GLO, Cr, Fe, TG and Ca. At an FPR of 7.7%, the detection rate increased to 54.5%, with an AUC^ROC^ of 0.839. The ROC curve for the SVM model is shown in Figure 3.

### 3.6. XGBoost Model

**Figure 4:**
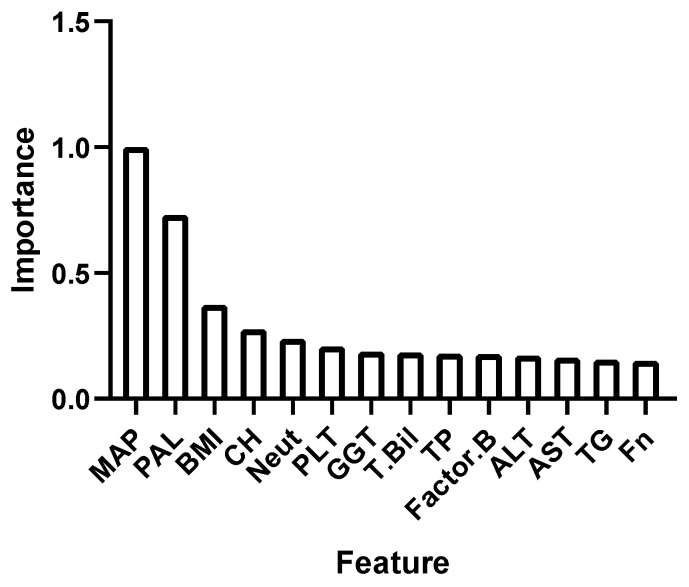
The importance of the features used for predicting late-onset preeclampsia by XGBoost.

**Figure 5:**
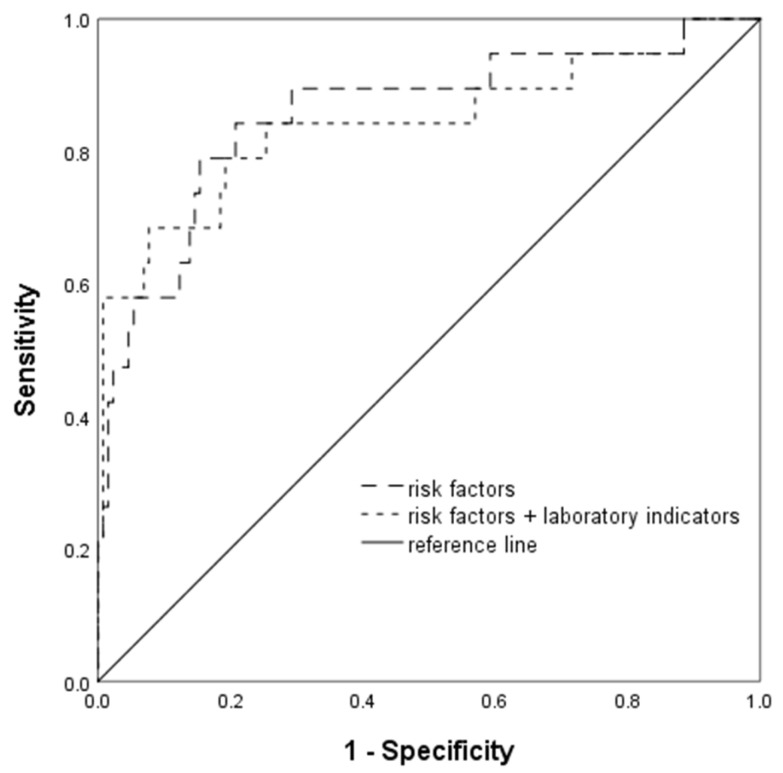
ROC curve of the XGBoost model.

First, the XGBoost model was established using only the risk factors and general data, achieving a detection rate of 31.6%, with a 1.5% FPR and an AUC^ROC^ of 0.857. Then, the laboratory indicators at 6–13 weeks of gestation were included in the model. The top 18 important indicators were MAP, PAL, BMI, chronic hypertension, Neu, PLT, GGT, T-Bil, TP, factor B, ALT, AST, TG, Fn, TIBC, Fe, LDL-C and P. The degree of importance of the features used for late-onset preeclampsia prediction is shown in Figure 4. At an FPR of 0.7%, the detection rate reached 52.60%, with an AUC^ROC^ of 0.842. The ROC curve of the XGBoost model is shown in Figure 5.

**Figure 6:**
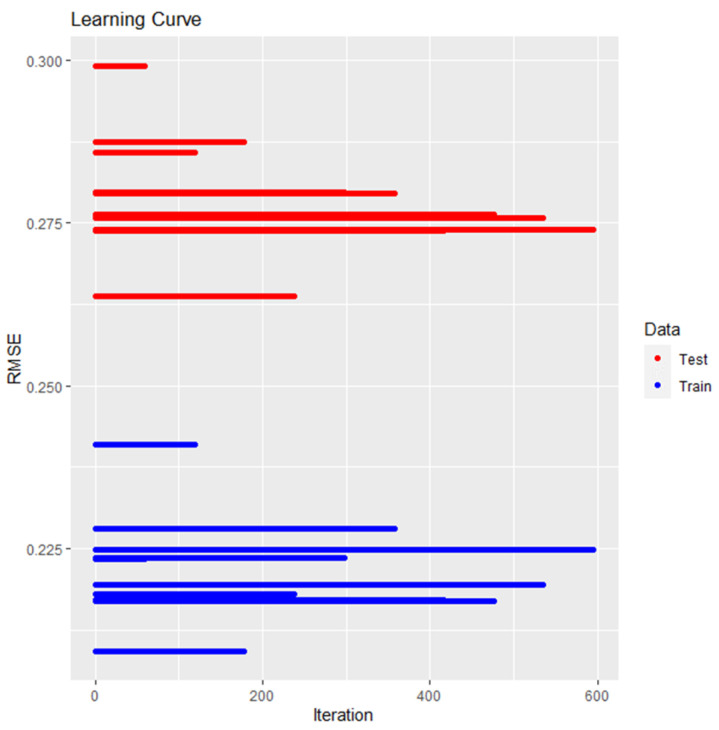
Learning curve of the RMSE for training and testing the XGBoost model. The x-axis represents the number of iterations, and the y-axis represents the RMSE value, which measures the prediction error, for both the training and testing sets. The curve shows how the RMSE changes as the number of iterations increases during model training.

To assess the prediction error, the root mean square error (RMSE) was utilized as a common measure. It represents the standard deviation of prediction errors. Figure 6 illustrates the learning curve of the RMSE for training and testing the XGBoost model. In this study, the training set’s predicted RMSE was concentrated around 0.20, with an increasing number of iterations, while the testing set was concentrated around 0.27. The model exhibited acceptable prediction accuracy and did not show significant overfitting.

The performances of three late-onset preeclampsia prediction models are showed in Table 5.

## 4. Discussion

### 4.1. Selection of Indicators for Prediction Models

The pathological manifestations of preeclampsia include abnormal invasion of trophoblasts into the uterine endometrium and incomplete remodeling of spiral arteries. The resulting placental ischemia leads to downstream effects, including endothelial dysfunction, vascular contraction, oxidative stress, and microthrombi, ultimately affecting multiple organ systems. Therefore, preeclampsia is characterized by the presence of hypertension, accompanied by proteinuria and/or maternal organ dysfunction, including liver dysfunction, acute kidney injury, hemolysis or thrombocytopenia [13,16]. Considering these, we selectively included proteinuria, liver function indicators, renal function indicators, iron metabolism indicators, D-dimer and the platelet count. Previous studies have shown that the neutrophil-to-lymphocyte ratio [17] and platelet-to-lymphocyte ratio [18] are helpful in predicting preeclampsia. Therefore, these two indicators were also included in our study. Significant differences were observed between the groups in terms of the liver function indicators, such as ALT, AST, ALP, GGT, and T-Bil, as well as the kidney function indicators, such as uric acid and CysC, proteinuria, and the platelet count. Other indicators, such as D-dimer, PLT/Lym, and sTfR, showed no statistically significant differences, which may be due to the lack of significant changes in these indicators in early pregnancy or their lack of significance for late-onset preeclampsia.

Studies have shown that women who develop preeclampsia have significantly elevated levels of lipid metabolism indicators compared to healthy pregnant women. These indicators include the total lipids, very low-density lipoprotein, triglycerides, and total fatty acids. Many of the observed metabolic differences in these women during early pregnancy are associated with overweight and obesity, representing the pre-pregnancy metabolic status [19]. In this study, the blood lipid indicators made important contributions to all three models.

Furthermore, as one of the mechanisms of PE, immune imbalance has gained increasing attention [20,21,22]. Inappropriate activation of the placental complement system can lead to placental dysfunction and subsequently contribute to the development of PE and other pregnancy complications [21]. Therefore, complement components and regulatory factors were also included in our model. The analysis results showed that the levels of C3, C4, C1q, factor B, and factor H in the PE group were higher than those in the control group. This indicates that complement indicators can be helpful in predicting PE in early pregnancy.

Fibronectin (Fn) is a key factor in cellular motility, and research [23] has demonstrated elevated expression of Fn in the placental cells of patients with preeclampsia. Fn inhibits migration and invasion of trophoblasts by influencing cellular motility and signaling. Aspirin can prevent preeclampsia by inhibiting Fn expression and reversing Fn-mediated trophoblast dysfunction. This indicator has shown significant importance in the early prediction of preeclampsia and has been included in all three models with high importance.

Hyperhomocysteinemia is a specific condition observed in the Han Chinese population. The TT genotype, derived from the C-T mutation in the key metabolic enzyme MTHFR’s C677T gene polymorphism, impedes the conversion of HCY into methionine, leading to hyperhomocysteinemia. The TT genotype accounts for approximately 25% of the Han Chinese population [24]. HCY may be associated with the pathogenesis of hypertensive disorders of pregnancy through vascular-mediated oxidative stress or direct damage [25]. However, in this study, during the gestational period of 6–13 weeks, the HCY levels in both the PE and control groups of pregnant women were within the normal reference range, and there were no significant differences between the groups. This may suggest that HCY has a greater impact on early-onset PE and a lesser impact on late-onset PE.

The maternal risk factors included in this study have already been shown to increase the risk of PE [12]. As Peking University Third Hospital serves as a critical care center for pregnant women in the Haidian district and is located in a region with advanced technology and culture, the women who seek medical care at our hospital are often high risk, highly educated, and of advanced maternal age. This, to some extent, weakens the predictive effects of factors such as age, nulliparity, assisted reproductive techniques, chronic kidney disease, and antiphospholipid syndrome on PE. It also impairs the predictive ability of other laboratory indicators of the occurrence of PE. Additionally, this explains the slightly higher incidence of PE (5.8%) in our study compared to the global incidence (2–4%) [1]. Furthermore, in this study, only two individuals in the PE group had antiphospholipid syndrome, and none had systemic lupus erythematosus. This may be due to the pregnant women with a predisposition to thrombotic diseases receiving continuous anticoagulant therapy in early pregnancy, effectively preventing abnormal blood flow and thrombus formation, thereby reducing the risk of PE.

### 4.2. Performance Comparison and Application of the Three Prediction Models

Because of the severe complications and adverse outcomes associated with early-onset PE, initial researchers paid more attention to it [26]. However, as our understanding of PE deepened, it was found that the majority of PE cases are late-onset PE. Previous studies have reported 82.7% [2] and 92% [19] of late-onset PE, respectively. Kenneth et al. conducted a retrospective study and found that among late-onset PE cases, 152 (57.6%) were diagnosed with severe PE, and at least one complication occurred in 81 cases (30.7%) [27]. Although this may be related to the study region being South Africa, it also emphasizes the importance of late-onset PE.

However, despite the recognition of its significance, predictive models for late-onset PE often underperform. Traditional markers, like the UtA-PI, PLGF, and PAPP-A, commonly used in early-onset PE prediction show limited utility for late-onset PE, with detection rates hovering around 45% at a 5% FPR [5].

Even in a study that included maternal characteristics, 8–12-week PAPP-A, free beta-human chorionic gonadotropin, blood pressure at 11–13^+6^ weeks, and UtA-PI, the detection rate was only 29.4% at a 5% FPR [6]. The above-mentioned models are all logistic regression models. In this study, a logistic regression model was also constructed as the baseline model. The results were consistent with previous studies. However, it should be noted that our study has a very low FPR and a very good negative predictive value, which can effectively predict the probability of pregnant women not developing late-onset PE in the future.

Each model in this study has its unique strengths and characteristics. Logistic regression is a simple and widely used method that is easy to understand and interpret. However, it can struggle when dealing with complex, nonlinear relationships and is limited by the events-per-variable principle, which restricts the number of variables that can be included in the model. Although logistic regression had a lower detection rate, it performed the best in terms of the AUC^ROC^, indicating that its predictions were the most reliable overall.

SVM is highly effective in handling high-dimensional data and has strong generalization ability, meaning it can work well with complex datasets. In this study, despite using 54 variables, SVM was able to incorporate all these factors into the model. However, the model’s performance was somewhat limited by the large dataset size and computational complexity. Although SVM achieved the highest detection rate of 54.5%, it also had the highest FPR, suggesting it is more sensitive but may generate more false positives.

XGBoost, based on gradient boosting decision trees, is particularly adept at handling nonlinear relationships and interactions between variables. This model has several advantages, such as the high predictive performance, the ability to handle high-dimensional and sparse features, and the strong interpretability. However, due to its complexity, XGBoost can suffer from overfitting. To mitigate this, we used the root mean square error (RMSE) to prevent overfitting while maintaining prediction accuracy. Among all three models, XGBoost showed a detection rate comparable to that of SVM, which is higher than the detection rates reported in the previous literature [5,6].

Finally, when comparing the impact of different variable combinations, we found that adding more variables generally increased the detection rate, as the model was able to use more information to make better predictions. However, it also led to an increase in the FPR, which may be due to the introduction of noise, causing the model to overfit. Therefore, while adding more data can improve accuracy, it is important to strike a balance to avoid overfitting and maintain model robustness.

### 4.3. Innovation and Limitations

Compared to previous studies, our research considered a more comprehensive set of variables and utilized more advanced prediction models, which may contribute to the better performance of our models. These models can be integrated into existing electronic health record systems to provide real-time risk assessments, allowing for earlier identification of high-risk pregnancies and timely intervention. Our model incorporated a wide range of routine laboratory indicators obtained during prenatal examinations, significantly reducing the need for expensive biomarkers or specialized equipment, thus making early detection of late-onset preeclampsia more feasible and effective in diverse clinical settings.

However, our study also has some limitations. Firstly, we focused on the early stages of pregnancy, and certain indicators might not exhibit significant changes during this period. Similar studies have employed machine learning algorithms to model late-onset PE, incorporating maternal factors and laboratory indicators from early and mid-pregnancy, achieving a detection rate of 77.1% at a 0.9% FPR [28]. Secondly, we did not consider other factors potentially influencing PE, such as lifestyle and genetic factors.

Overall, future research should aim to integrate a broader range of variables, including genetic data, lifestyle factors, and mid-pregnancy indicators, to improve the clinical applicability and predictive power of models for late-onset PE.

In conclusion, after incorporating laboratory indicators, all three models demonstrated an improvement in performance compared to when only risk factors were included. Relative to the logistic regression model, both the SVM and XGBoost models significantly improved the detection rates for late-onset preeclampsia, albeit with the SVM model displaying a higher FPR. Both the logistic regression and XGBoost models exhibited higher negative predictive values of 99.3%, highlighting their capacity to accurately identify pregnant women who are unlikely to develop late-onset preeclampsia.

## Figures and Tables

**Table 1:** General information and risk factors of the control group and PE group.

Variables	Control Group (n = 1618)	PE Group (n = 110)	Statistical Magnitude	*p* Value
Age (year)	33 ± 4	34 ± 3	−1.821	0.069
BMI (kg/m^2^)	21.8 ± 2.9	24.6 ± 3.7	−7.872	<0.001 *
MAP (mmHg)	84 ± 9	93 ± 10	−11.119	<0.001 *
Risk factors [n(%)]				
Chronic hypertension	7 (0.4)	21 (19.1)		<0.001 *
Diabetes	20 (1.2)	6 (5.5)		0.002 *
Systemic lupus erythematosus (SLE)	3 (0.2)	0 (0)		1.000
Antiphospholipid syndrome (APS)	19 (1.2)	2 (1.8)		0.883
Kidney disease	10 (0.6)	3 (2.7)		0.045 *
Assisted reproductive technology	300 (18.5)	33 (30)		0.003 *
Nulliparity	1175 (72.6)	92 (83.6)		0.011 *

* *p* values were statistically different, *p* < 0.05.

**Table 2:** The laboratory indicators at 6–13 weeks of gestation of the control group and PE group.

Variables	Control Group (n = 1618)	PE Group (n = 110)	Statistical Magnitude	*p* Value
Liver function indicators				
ALT (U/L)	12 [10, 18]	17 [12, 26]	−6.012	<0.001 *
AST (U/L)	18 [16, 21]	20 [17, 24]	−3.523	<0.001 *
TBA (μmol/L)	2 ± 2.3	1.8 ± 1.3	0.674	0.500
TP (g/L)	73.9 ± 4.1	73.5 ± 4.5	1.016	0.310
Alb (g/L)	43.4 ± 2.4	43.3 ± 2.8	0.359	0.720
GLO (g/L)	31 ± 3	30 ± 3	1.111	0.267
LDH (U/L)	169 ± 31	167 ± 34	0.572	0.568
ALP (U/L)	52 ± 13	56 ± 15	−2.952	0.003 *
GGT (U/L)	14 [11, 17]	17 [14, 28]	−7.568	<0.001 *
D-Bil (μmol/L)	1.3 ± 0.6	1.3 ± 0.6	1.016	0.310
T-Bil (μmol/L)	13.5 ± 4.5	11.9 ± 4	3.589	<0.001 *
PAL (mg/L)	212 ± 35	225 ± 58	−3.526	<0.001 *
Renal function indicators				
Uric acid (μmol/L)	217 ± 48	242 ± 61	−4.136	<0.001 *
Urea (mmol/L)	3.2 ± 0.8	3.2 ± 0.7	0.815	0.417
Cr (μmol/L)	60 ± 7	61 ± 7	−0.567	0.571
Ca (mmol/L)	2.43 ± 0.09	2.43 ± 0.1	0.028	0.977
P (mmol/L)	1.26 ± 0.13	1.25 ± 0.14	0.942	0.346
CysC (mg/L)	0.56 ± 0.1	0.58 ± 0.09	−2.696	0.007 *
Blood lipid indicators				
TG (mmol/L)	1.03 ± 0.47	1.29 ± 0.59	−4.502	<0.001 *
CHO (mmol/L)	4.24 ± 0.9	4.53 ± 1.53	−1.915	0.058
HDL-C (mmol/L)	1.54 ± 0.29	1.46 ± 0.3	2.832	0.005 *
LDL-C (mmol/L)	2.17 ± 0.57	2.31 ± 0.63	−2.240	0.027 *
Lp(a) (mg/L)	58 [30, 135]	55 [22, 83]	−2.250	0.024 *
ApoA1 (g/L)	1.52 ± 0.3	1.51 ± 0.32	0.389	0.697
ApoB (g/L)	0.64 ± 0.15	0.71 ± 0.18	−3.924	<0.001 *
ApoE (g/L)	35 ± 8.7	38 ± 9.3	−3.461	0.001 *
Complement/inflammatory markers				
C3 (g/L)	122 ± 21	132 ± 23	−4.825	<0.001 *
C4 (g/L)	23.24 ± 7.21	25.44 ± 7.42	−3.096	0.002 *
C1q (mg/L)	195 ± 36	199 ± 35	−1.333	0.183
Factor B (mg/L)	324 ± 39	342 ± 41	−4.504	<0.001 *
Factor H (mg/L)	312 ± 52	338 ± 55	−4.973	<0.001 *
US-CRP (mg/L)	0.80 [0.43, 1.74]	1.78 [0.74, 3.52]	−5.265	<0.001 *
Blood cell count				
PLT (×10^9^/L)	242 ± 49	263 ± 63	−3.420	0.001 *
Neu (×10^9^/L)	5.33 ± 1.81	6.22 ± 2.82	−3.249	0.002 *
Lym (×10^9^/L)	1.88 ± 0.45	2.02 ± 0.52	−3.015	0.003 *
PLT/Lym	133.68 ± 33.98	137.75 ± 42.97	−0.972	0.333
Neu/Lym	2.94 ± 1.02	3.17 ± 1.13	−2.283	0.023 *
Iron metabolism				
Fe (μmol/L)	22 ± 6.7	20.5 ± 6.5	2.226	0.026 *
TIBC (μmol/L)	60.6 ± 8.5	65.3 ± 9.3	−5.460	<0.001 *
UIBC (μmol/L)	39 ± 12	45 ± 11	−5.381	<0.001 *
sTfR (%)	27.49 ± 13.19	27.42 ± 9.96	0.056	0.955
Others				
Proteinuria	2 (0.1)	7 (6.4)		<0.001 *
Fn (mg/L)	204 ± 37	223 ± 41	−5.172	<0.001 *
HCY (μmol/L)	6.5 ± 1.7	6.6 ± 1.9	−0.722	0.470
D-dimer (mg/L)	0.15 [0.15, 0.15]	0.15 [0.15, 0.15]	−1.044	0.296

* *p* values were statistically different, *p* < 0.05. Alanine aminotransferase (ALT), aspartate aminotransferase (AST), total bile acid (TBA), total protein (TP), albumin (Alb), globulin (GLO), lactate dehydrogenase (LDH), alkaline phosphatase (ALP), glutamyl transferase (GGT), direct bilirubin (D-Bil), total bilirubin (T-Bil), prealbumin (PAL), creatinine (Cr), cystatin C (CysC), triglycerides (TG), cholesterol (CHO), high-density lipoprotein cholesterol (HDL-C), low-density lipoprotein cholesterol (LDL-C), lipoprotein a (Lp(a)), apolipoprotein A1 (ApoA1), apolipoprotein B (ApoB), apolipoprotein E (ApoE), ultrasensitive C-reactive protein (US-CRP), platelet count (PLT), neutrophil count (Neu), lymphocyte count (Lym), total iron-binding capacity (TIBC), unsaturated iron-binding capacity (UIBC), soluble transferrin receptor (sTfR).

**Table 3:** Logistic regression model established using general information and risk factors.

Variables	B	Significance	Exp (B)	95% Confidence Interval of Exp (B)
Chronic hypertension	2.561	<0.001 *	12.943	(4.577, 36.599)
Diabetes	0.763	0.210	2.144	(0.651, 7.058)
APS	0.554	0.549	1.740	(0.285, 10.625)
Kidney disease	1.340	0.137	3.818	(0.653, 22.336)
Assisted reproductive technology	0.099	0.710	1.104	(0.655, 1.862)
Nulliparity	0.943	0.005 *	2.568	(1.335, 4.942)
Age	0.284	0.021 *	1.328	(1.044, 1.691)
BMI	0.588	<0.001 *	1.800	(1.483, 2.183)
MAP	0.810	<0.001 *	2.247	(1.751, 2.883)
Constant	−4.187	<0.001	0.015	

* *p* values were statistically different, *p* < 0.05.

**Table 4:** Logistic regression model established by selecting 15 factors encompassing general information, risk factors, and laboratory indicators during 6–13 weeks of pregnancy.

Variables	B	Significance	Exp(B)	95% Confidence Interval of Exp(B)
Chronic hypertension	2.757	<0.001 *	15.751	(5.314, 46.688)
Nulliparity	0.948	0.005 *	2.582	(1.323, 5.039)
Age	0.339	0.009 *	1.403	(1.088, 1.811)
BMI	0.475	<0.001 *	1.608	(1.274, 2.03)
MAP	0.826	<0.001 *	2.284	(1.75, 2.979)
Proteinuria	3.839	<0.001 *	46.464	(7.157, 301.657)
LDH	−0.389	0.003 *	0.678	(0.524, 0.878)
CHO	0.968	0.002 *	2.631	(1.438, 4.816)
LDL-C	−1.094	<0.001 *	0.335	(0.182, 0.616)
ApoA1	−0.425	0.017 *	0.654	(0.461, 0.926)
UIBC	0.352	0.005 *	1.422	(1.115, 1.814)
Factor B	−0.493	0.037 *	0.611	(0.385, 0.97)
Fn	0.480	0.044 *	1.616	(1.013, 2.576)
Neu	0.426	<0.001 *	1.531	(1.213, 1.932)
GGT	0.417	<0.001 *	1.517	(1.224, 1.88)
Constant	−4.509	<0.001 *	0.011	

* *p* values were statistically different, *p* < 0.05.

**Table 5:** Performance comparison of three late-onset preeclampsia prediction models.

Model	Indicator	FPR	Detection Rate	AUC^ROC^
Logistic regression	Risk factors	0.4%	19.1%	0.833
	Risk factors + laboratory indicators	0.7%	27.3%	0.877
SVM	Risk factors	0.0%	27.3%	0.645
	Risk factors + laboratory indicators	7.7%	54.5%	0.839
XGBoost	Risk factors	1.5%	31.6%	0.857
	Risk factors + laboratory indicators	0.7%	52.6%	0.842

## Data Availability

Data is contained within the article.
